# A dense longitudinal multimodal single-subject rs-fMRI dataset acquired by self-administered scanning

**DOI:** 10.1038/s41597-026-06879-z

**Published:** 2026-02-21

**Authors:** Evgeny D. Petrovskiy

**Affiliations:** International Tomography Center SB RAS, 630090 Novosibirsk, Russia

## Abstract

Dense longitudinal neuroimaging usually requires substantial institutional resources, yet can also be achieved by an individual using standard clinical MRI infrastructure. This work presents a multimodal single-subject dataset comprising 85 hours of resting-state fMRI acquired over 11 months, including 51.6 hours under a standardized protocol (paired eyes-open/-closed runs, 128 sessions over 7.5 months). Additional data include 195 T1-weighted structural scans, 54 diffusion MRI sessions, physiological recordings, pre-session behavioral assessments, and detailed medication and lifestyle logs. Scans were collected primarily via self-administered acquisition on a clinical 3 T system, with sub-3 mm between-session positioning reproducibility observed in later sessions. Quality control identified 58 hours of low-motion data (mean framewise displacement <0.2 mm), with higher-motion runs occurring predominantly during sleep. The acquisition period included antidepressant dose changes and seasonal variation, forming a single-subject naturalistic context with collinear factors that preclude causal inference. The dataset follows the BIDS standard and is intended for methodological development, reliability analyses, preprocessing benchmarking, and educational use.

## Background & Summary

Longitudinal neuroimaging studies provide unique insights into individual neural variability and state-dependent connectivity patterns that are obscured by between-subject heterogeneity in traditional cross-sectional designs. Large-scale initiatives such as the Human Connectome Project^1^ and UK Biobank^2^ have generated extensive multimodal datasets through substantial infrastructure investments and multi-institutional collaborations, but their predominantly cross-sectional or sparse longitudinal designs are limited for investigation of individual temporal dynamics. Dense temporal sampling within single individuals offers complementary insights into within-person neural variability. Pioneering single-subject intensive longitudinal studies include the MyConnectome project^3^ (106 sessions over 18 months, ~14–16 hours of fMRI data) and the Kirby Weekly dataset^4^ (158 sessions over 3.5 years, ~18 hours of fMRI data) which established methodological frameworks for within-individual neuroimaging research. However, these efforts required dedicated research infrastructure, specialized personnel, and sustained institutional support.

**Fig. 1:**
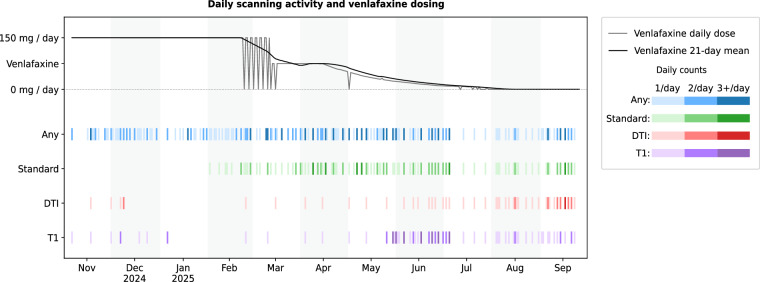
Daily scanning activity and venlafaxine dosing across the 11-month study. Each rectangle represents the number of sessions per day for the indicated modality: any session (blue), standardized protocol sessions (green), diffusion MRI (red), and structural T1-weighted imaging (purple). Color intensity encodes 1, 2, or 3 + sessions per day. The gray line above shows the daily venlafaxine extended-release dose, with the black line indicating the 21-day rolling mean. This visualization summarizes data collection density and medication taper timeline on a unified time axis; the timeline illustrates temporal co-occurrence rather than separable experimental factors.

This dataset demonstrates that individual researchers can conduct neuroimaging studies matching quality standards of dedicated research facilities. The study comprises 85 hours of resting-state fMRI collected across 11 months (321 days), including 51.6 hours from 128 standardized protocol sessions during a 7.5-month period (233 days) (Fig. 1).

The standardized protocol consisted of paired 10-minute eyes-open and 14-minute eyes-closed acquisitions, preceded by a 3-month development phase optimizing self-scanning procedures and acquisition parameters. The majority of scanning sessions (125 of 128 standard protocol sessions) were conducted using self-administered protocols without operator presence on a clinical 3 T system with standard sequences.

The acquisition period included a planned antidepressant (venlafaxine) taper and almost parallel seasonal change during the standardized data collection phase. These factors - pharmacological state, seasonal photoperiod, and procedural learning - evolve on the same time axis and are therefore strongly collinear. Because they cannot be disentangled within a single-subject design, the dataset is not intended to support causal inference about medication, seasonality, or their neural effects. Instead, these variables provide a rich but confounded naturalistic context accompanying the primary methodological contribution: dense self-administered longitudinal neuroimaging.

The dataset includes 195 structural T1-weighted scans, 54 diffusion tensor imaging sessions, detailed medication dosing logs, pre-session psychological assessments (PANAS, Psychomotor Vigilance Task), physiological monitoring (respiratory and cardiac) for 89 sessions, and daily lifestyle logs (sleep timing, caffeine and alcohol consumption, exercise, step counts).

Quality control assessment revealed 58 hours of data meeting strict motion criteria (mean framewise displacement < 0.2 mm) and 75.4 hours at mean FD < 0.3 mm. In the subset of sessions where vigilance state was systematically recorded (90% of standard protocol runs, 64% overall), motion characteristics varied systematically: eyes-open/awake sessions (34.1 hours) showed excellent low motion (mean FD 0.13 mm), eyes-closed/awake (10 hours, 0.18 mm) and partial sleep (7.3 hours, 0.20 mm) showed moderate increases, while frank sleep (17.8 hours, 0.29 mm) exhibited higher but physiologically expected motion. This distribution enables subsetting by vigilance/motion for sensitivity analyses and methodological comparisons.

No field maps or reversed phase-encoding acquisitions were obtained; functional and diffusion data are therefore provided without susceptibility distortion correction. This limits absolute spatial accuracy and anatomical localization, particularly in ventral frontal and temporal regions.

Overall, this dataset provides an unusually dense multimodal single-subject timeseries suitable for methodological development, reliability analyses, preprocessing and QC benchmarking, and teaching/demonstration purposes, while explicitly documenting the constraints typical of clinical acquisition environments. While more advanced acquisition techniques could in principle be incorporated under appropriate institutional oversight, the present dataset is intended to demonstrate feasibility and data characteristics within standard clinical settings rather than to prescribe a transferable scanning protocol.

## Methods

### Participant characteristics & timeline

#### Participant characteristics

The participant was a 34-year-old male researcher with extensive prior fMRI research experience in both investigator and participant roles. At study initiation, the participant was receiving stable antidepressant therapy (venlafaxine extended-release, 150 mg/day) for over 12 months. The participant had no contraindications to MRI scanning, no history of claustrophobia or movement disorders, and had demonstrated tolerance for extended scanning sessions in previous studies.

#### Timeline and study phases

Data collection spanned 11 months (November 2024 – September 2025) with three distinct phases (Fig. 1):

**Phase 1 - Protocol Development** (November 2024 – January 2025, 3 months): Initial scanning sessions began in November 2024, with transition to self-administered scanning in December 2024. This phase involved optimization of scan protocols, self-scanning procedures, and data collection workflows. High geometric variability characterized this phase before standardization of positioning techniques.

**Phase 2 - Initial Standardized Protocol** (February – early June 2025, 4 months): Implementation of standardized acquisition protocols concurrent with initiation of gradual antidepressant discontinuation. While scan protocols were consistent during this phase, geometric variability remained elevated due to reliance on non-anatomical positioning references.

**Phase 3 - Optimized Protocol with Enhanced Positioning** (June 10 – September 2025, 3.5 months): Introduction of laser-crosshair-to-eyes positioning technique (June 10, 2025) achieved substantial reduction in between-session geometric variability (translation SD < 3 mm, rotation SD < 1.5°). This improved positioning reproducibility, combined with increased procedural familiarity, enabled more frequent inclusion of T1-weighted and diffusion tensor imaging acquisitions. Mid-phase evaluation of T1-weighted slice thickness (1.0–2.0 mm in 0.25 mm increments) resulted in adoption of 1 mm protocol for optimal anatomical detail.

#### Medication timeline

Venlafaxine extended-release dosing was maintained at 150 mg/day through February 2025, providing a stable baseline period. Capsules were taken consistently each morning, but exact administration times were not logged as daily variability was clinically negligible.

The tapering process involved three distinct phases:

**Initial irregular dosing** (February 23 – March 12, 2025): 150 mg capsules at ~36-hour intervals in a morning-evening-skip pattern, which resulted in withdrawal symptoms likely due to extended-release formulations being optimized for 24-hour dosing.

**Stabilization period** (March 13 – April 15, 2025): Approximate capsule halves (~75 mg daily) to re-establish consistent plasma levels.

**Gradual exponential reduction** (April 16 – last administration July 24, 2025): Progressive taper with fine-grained sub-capsule dose adjustments, following an approximately exponential schedule with dose halved about every 4 weeks, and tolerated without major adverse events.

Importantly, the taper period partially overlaps in time with two other evolving factors in this study: seasonal changes in daylight (spring–summer) and procedural improvements in self-administered scanning. These factors are therefore temporally correlated and cannot be separated as independent causal drivers of longitudinal variation. We explicitly treat medication and other time-varying covariates as descriptive/contextual variables and address their co-occurrence in the Technical Validation analyses.

A complete log of medication administration times and doses is included in derivatives/medication/ for all tapering phases. During the stabilization and exponential reduction periods, typical day-to-day variation in administration timing was approximately ±3 hours.

### Ethics statement

This longitudinal single-subject study was approved by the Ethics Committee of the International Tomography Center SB RAS (approval No. 4, October 24, 2024). The author served as the sole participant, and the dual role of investigator and participant was explicitly reviewed as part of the ethics approval, including potential conflicts of interest and participant safety. Medical suitability for MRI participation was confirmed within the framework of standard MRI safety practices applied at the institution, and no contraindications were identified. Written informed consent was obtained for participation and for public sharing of anonymized data. All procedures conformed to institutional MRI safety standards and the Declaration of Helsinki.

### Safety framework for self-administered scanning

Self-administered MRI scanning entails safety considerations beyond standard participant workflows and was conducted only under explicit institutional approval. The ethics committee and MRI facility reviewed and approved the self-administered workflow as part of the study protocol, including risk assessment related to autonomous table positioning, scanner operation without direct operator presence, and the author’s dual role as investigator and participant.

All acquisitions relied exclusively on manufacturer-certified scanner controls and built-in safety systems; no hardware interlocks were bypassed and no modifications to standard safety mechanisms were made. Scanning was performed during staffed facility hours, with institutional emergency procedures continuously applicable.

The participant had extensive prior experience in MRI research environments, no contraindications to MRI, and completed institutional MRI safety training. No adverse events occurred during the study.

This self-administered scanning approach is not transferable to other participants or settings and must not be interpreted as a recommended or broadly applicable scanning paradigm. Replication of similar procedures should not be attempted without formal institutional safety review and approval. The dataset documents feasibility under controlled institutional conditions and does not endorse unsupervised or routine self-administered MRI use.

### MRI Acquisition

All scans were acquired using a 3.0 Tesla Philips Ingenia scanner with a 16-channel head coil at the Center of Collective Use “Mass Spectrometric Investigations” SB RAS, International Tomography Center, Novosibirsk, Russian Federation. Data collection occurred over 11 months during which the scanning protocol evolved from initial development to a standardized self-administered workflow.

#### Functional MRI

Resting-state functional MRI data were collected using two acquisition protocols that differed in spatial resolution and brain coverage.

Due to scanner software constraints on the total number of slices per run, the maximum duration of a single acquisition depended on slice count. Standardized run durations were selected to remain within these limits. A small number of longer acquisitions were obtained during a temporary expansion of software licensing.

##### Standard sequence (3iso)

Single-shot gradient echo echo-planar imaging (EPI) with a repetition time (TR) = 2500 ms, and echo time (TE) = 31.5 ms, flip angle = 90°, Spectral Presaturation with Inversion Recovery (SPIR) fat suppression, with field of view (FOV) = 220 × 220 mm^2^, 3 mm isotropic voxel size acquired on 76 × 73 matrix and reconstructed to 112 × 112 matrix (2 mm in-plane resolution), 48 axial slices with 10% slice gap (0.3 mm), SENSitivity Encoding (SENSE) parallel imaging acceleration factor = 3.0. This protocol provided full brain coverage with tolerance for head positioning variations during self-scanning. Ten dummy scans were acquired and automatically discarded by the scanner before saved volumes.

##### Standard protocol definition

The core dataset consists of paired acquisitions using the 3iso sequence: (1) a 240-volume (10-minute) eyes-open run not preceded by any eyes-closed acquisition within the same session, followed by (2) a 340-volume (14-minute) eyes-closed run. This 24-minute paired protocol constituted the primary data collection approach across 128 sessions, totaling 51.6 hours of standardized resting-state data.

##### High-resolution protocol (2p5iso)

Early sessions employed higher spatial resolution with TR = 2500 ms, TE = 35 ms, 2.5 mm isotropic voxel size, 42 axial slices with no gap providing partial brain coverage, SENSE factor = 2.0. Ten dummy scans were acquired and automatically discarded.

This protocol, with reduced brain coverage along the superior–inferior direction, proved insufficiently robust to positioning variability during self-scanning and was replaced by the 3iso sequence, which provided consistent full-brain coverage.

#### Structural and diffusion MRI

##### T1-weighted imaging

Three-dimensional gradient echo sequence with TR = 7.6 ms, TE = 3.7 ms, inversion time (TI) = 906 ms, flip angle = 8°, FOV = 250 × 250 mm^2^. The in-plane resolution was 0.87 × 0.87 mm^2^. Several sessions tested slice thicknesses (1.0–2.0 mm in 0.25 mm increments) to optimize image quality while maintaining consistent in-plane resolution and vertical field of view.

##### Acquisition evolution

Data collection began with 2.0 mm nominal slice thickness. Midway through the study, a systematic slice-thickness evaluation was performed – 13-14 sessions each at 1.0, 1.25, 1.5, 1.75, and 2.0 mm – to determine the optimal trade-off between scan duration, signal-to-noise ratio, and segmentation reliability while maintaining constant vertical field of view. Accordingly, the number of slices was adjusted with thickness (e.g., 181 slices at 2.0 mm vs. 362 slices at 1.0 mm) to preserve anatomical coverage. Following this evaluation, the protocol was finalized at 1.0 mm slice thickness for all subsequent sessions.

All T1-weighted scans were reconstructed with −50% slice gap, meaning intermediate slices were interpolated at half-step positions (e.g., 1.0 mm slices positioned at 0.5 mm intervals, 2.0 mm slices at 1.0 mm intervals). File headers correctly report this slice spacing in the voxel dimensions, which accurately reflects geometric positioning but not independent spatial resolution of the acquisition.

All T1-weighted structural images were defaced prior to public release using *pydeface* v2.0.0 (https://github.com/poldracklab/pydeface)^5^ to remove facial features while preserving brain anatomy.

##### Diffusion tensor imaging (DTI)

Single-shot spin-echo EPI sequence with 32 directions, b-values of 0 and 800 s/mm², TR = 5012 ms, TE = 94 ms, flip angle = 90°, FOV = 224 × 224 mm², 2 mm isotropic voxel size (acquired at 112 × 110 matrix; reconstructed to 128 × 128, 1.75 mm in-plane resolution), 60 or 75 slices, SENSE factor = 2.0. Slice count variation reflected minor field-of-view adjustments.

#### Self-scanning procedure

The majority of scanning sessions (125 of 128 standard protocol sessions; 189 of 243 total sessions) were conducted without operator presence using a self-administered approach in which the participant initiated predefined scan protocols and controlled table positioning while already positioned on the scanner bed. No novel hardware modifications were introduced, and all acquisitions used standard clinical scanner interfaces and manufacturer-provided safety systems.

Detailed operational steps are intentionally omitted, as the procedure was implemented under institutional safety approval and is not intended as a generalizable or participant-facing protocol.

During later phases of the study, between-session positioning reproducibility was improved by adopting a simple, visually accessible anatomical reference for head alignment that could be consistently identified by the participant while positioned on the scanner bed. Specifically, scanner laser crosshairs were aligned relative to the eye position, providing a stable and repeatable reference point without reliance on external operators or anatomical landmarks not visible from within the bore. This reference-based approach was used solely to improve longitudinal consistency of positioning and is described here at a conceptual level. The effectiveness of this positioning strategy is evaluated quantitatively in the Technical Validation section.

#### Physiological monitoring

For a subset of scanning sessions (89 sessions, 170 functional runs), simultaneous physiological data were recorded using the scanner’s integrated respiratory belts and pulse plethysmography sensors. Physiological data were stored as SCANPHYSLOG files and converted to BIDS-compliant _physio.tsv.gz format with accompanying JSON metadata using custom Python scripts. Because SCANPHYSLOG files did not contain reliable per-volume triggers, volume triggers were synthesized by detecting gradient-active periods from the gradient channel envelope and calculating backwards from the acquisition endpoint using known TR and volume counts.

### Quality control

Run-level quality metrics were derived using MRIQC v25.1.0 deployed as a Docker container. Complete outputs including framewise displacement (FD), DVARS, temporal signal-to-noise ratio (tSNR), and other image quality metrics are provided in derivatives/mriqc/. All quantitative summaries were derived from MRIQC’s per-run JSON and TSV outputs using custom Python scripts that aggregate metrics across sessions and produce condition-specific and longitudinal quality summaries. Processing code is available in the project repository.

### Behavioral and physiological assessments

#### Pre-scanning psychological assessments

For the majority of sessions (111 of 128 standard protocol sessions; 146 of 243 total sessions), pre-session assessments were conducted using a self-developed web application immediately before scanning. Assessments included the Positive and Negative Affect Schedule (PANAS) and a 3-minute Psychomotor Vigilance Task (PVT).

The PANAS^6^ consists of 20 mood adjectives rated on a 5-point scale, yielding positive affect (10 items: interested, excited, strong, enthusiastic, proud, alert, inspired, determined, attentive, active) and negative affect (10 items: distressed, upset, guilty, scared, hostile, irritable, ashamed, nervous, jittery, afraid) subscale scores ranging from 10 to 50.

The PVT required participants to respond to visual stimuli appearing at random intervals (2–10 seconds) by pressing a key as quickly as possible. Standard summary metrics were computed: median reaction time (robust measure of typical alertness) and lapse count (number of responses >500 ms, indicating attention failures).

Session-level summaries (median RT, lapse count, PANAS positive/negative scores) are provided in sub-001_sessions.tsv for direct use as covariates. Raw trial-level PVT data and item-level PANAS responses are preserved in sourcedata/assessments/ for validation and alternative scoring approaches.

#### In-scanner alertness monitoring

Subjective alertness was assessed using the Stanford Sleepiness Scale on a per-run basis. Scores were either memorized or written on paper during brief inter-run intervals. When alertness changed during a run, both start and end values were recorded (e.g., “3..5”).

No instruction to sleep was given; any sleep episodes were incidental and recorded for transparency. Sleep occurrence was categorized as “No,” “Yes” (sleep covering most of run duration), or “A little” (sleep covering smaller portion).

These ratings are coarse, subjective estimates intended for covariate modeling or censoring rather than objective vigilance measures.

#### Medication logging and pharmacological regressors

Venlafaxine administration was tracked throughout the study using a private Telegram chat enhanced with Apple Shortcuts automation for standardized entries.

For the pre-taper baseline period (stable 150 mg/day dosing), exact administration times were not recorded as capsules were consistently taken each morning. To support pharmacokinetic modeling, daily dose events at 08:00 were synthetically generated for this period and are explicitly flagged (source = “generated”) in the raw logs, while all hand-entered events during the taper are marked as source = “logged”.

The method of dose preparation is recorded for each administration as dose_type with three levels: full capsule, capsule half, and bead-counted dosing. For bead-counted doses, the logs retain the bead count and the beads-per-capsule constant used for mg conversion (avg_beads_per_capsule), along with the original calculation expression (raw_dose_calculation), for full traceability.

##### Pharmacological covariates

To provide descriptive temporal annotations without implying mechanistic interpretability, we computed several dose-related variables intended solely as metadata for temporal alignment and exploratory filtering:

1. **Most recent dose amount** (mg) within 48-hour window prior to scanning (acute exposure proxy).
2. **21-day rolling average dose**, a smoothed exposure metric reflecting longer clinical timescales.
3. **Acute withdrawal**, a dose-weighted indicator for periods >28 hours since last administration during active dosing.
4. **Withdrawal burden**, quantifying accumulated physiological stress from irregular dosing patterns with exponential decay (10-day half-life) that continues after the final dose.

Complete derivation methods and reproducible Python code are provided in code/calculate_vfx_regressors.py with detailed documentation in accompanying metadata.

#### Lifestyle and behavioral logging

Daily events and behaviors were logged using a private Telegram chat system. The participant captured sleep timing, caffeine consumption, physical activity, and alcohol intake with minimal burden.

Sleep journaling: Sleep onset and offset times were typically entered as intervals after waking, with 91% coverage during standardized protocol sessions (86% overall).

Coffee intake was recorded in cups prepared with a moka pot or home espresso machine, both consistently prepared by the participant with similar amounts of ground coffee.

Alcohol consumption was logged in approximate 0.5 L beer-equivalent units (5% ABV).

Exercise tracking captured intentional workout sessions (structured resistance training, dedicated treadmill sessions) throughout the study.

Step counts were captured via Apple Health records integrated with iPhone accelerometry.

## Data Records

The complete dataset is organized according to the Brain Imaging Data Structure (BIDS) specification and is publicly available at OpenNeuro (dataset accession number: ds007328, 10.18112/openneuro.ds007328.v1.0.0)^7^. All data correspond to a single participant (sub-001) across 243 scanning sessions conducted over 321 days (November 2024 – September 2025).

### Dataset organization and session naming

Sessions are identified using the naming convention ses-YYYYMMDDNN or ses-YYYYMMDDNNstd, where YYYYMMDD represents the acquisition date and NN indicates the session order within that day (typically 01 or 02). Sessions containing the standardized protocol (240-volume eyes-open 3iso run without preceding eyes-closed acquisition, followed by 340-volume eyes-closed 3iso run) are marked with the std suffix. Of the 243 total sessions, 128 sessions are marked with std, representing the core longitudinal dataset collected over 7.5 months.

Complete session-level metadata, including acquisition timing, modality counts, protocol indicators, physiological monitoring status, and pre-session assessment completion, are provided in sub-001/sub-001_sessions.tsv with comprehensive variable definitions in the accompanying JSON sidecar (see Supplementary Materials).

### Functional MRI data

Resting-state fMRI data comprise 458 runs totaling 85 hours across 230 sessions. All functional data are located in session-specific func/ subdirectories with the naming pattern:

sub-001_ses-YYYYMMDDNN(std)_task-rest_acq- < PROTOCOL > _run- < N > _bold.nii.gz

The acq- field encodes spatial resolution (2p5iso or 3iso), eyes condition (EO/EC), and protocol membership (std suffix for standardized protocol runs).

### Standard protocol data (3iso sequence)

A total of 415 runs were acquired using the 3 mm isotropic sequence. Of these, 256 runs (51.6 hours across 128 sessions over 233 days) constitute the standardized paired acquisition: 128 eyes-open runs (240 volumes, 10 minutes) and 128 eyes-closed runs (340 volumes, ~14 minutes). The remaining 159 runs include shorter developmental acquisitions (63 runs, 120–200 volumes, 5–8.5 minutes) and intermediate variants (91 runs, 201–341 volumes, up to 14 minutes) used to test sequence parameters and reconstruction settings. A small subset of five extended runs (342–1018 volumes, 14–42 minutes) was collected during a period of expanded licensing (December 2024 – January 2025). All but one of these extended 3iso runs were self-administered.

### High-resolution protocol data (2p5iso sequence)

An additional 43 runs from the early developmental phase used a 2.5 mm isotropic protocol with nominally partial coverage (42 slices, no gap). Several of these runs approached or reached upper limit of run duration (up to 390 volumes, ~16 minutes), and a few extended runs acquired under expanded licensing reached 469–887 volumes (19–37 minutes). Extended 2p5iso runs were operator-controlled, ensuring stable positioning, and visual inspection confirmed adequate brain coverage.

#### Physiological monitoring

Simultaneous respiratory and pulse plethysmography recordings are available for 89 sessions (170 functional runs, including 73 standard protocol sessions). Data are provided as BIDS-compliant *_physio.tsv.gz files with three columns: respiratory signal (resp), pulse plethysmography (ppu), and synthesized volume triggers (trigger). Metadata including sampling frequency (500 Hz) and acquisition parameters are documented in accompanying JSON sidecars.

#### Run-level metadata

A dataset-wide summary file (derivatives/sub-001_func_runs.tsv) consolidates all functional run metadata, including session and run identifiers, sequence protocol, volume counts, eyes condition, standardized protocol indicators (stdEO, stdEC), self-reported sleep occurrence, and Stanford Sleepiness Scale ratings (sleepiness_start, sleepiness_end, sleepiness_mean). This file enables efficient filtering and quality-based subsetting for analysis. Complete variable definitions are provided in sub-001_func_runs.json.

### Structural and diffusion MRI Data

#### T1-weighted imaging

A total of 195 three-dimensional T1-weighted scans were acquired across 84 sessions. Several sessions contained multiple T1 runs - either repeats within the same session to permit potential test-retest analyses, or protocol variants collected during mid-study sequence updates. These additional acquisitions provide opportunities to evaluate short-term reproducibility of structural MRI metrics and assess how slice-thickness differences influence downstream processing outcomes.

Structural data are stored in session-specific anat/ subdirectories using the naming pattern sub-001_ses-YYYYMMDDNN(std)_acq- < THICKNESS > _T1w.nii.gz, where acq- encodes nominal slice thickness (1 mm, 1p25mm, 1p5mm, 1p75mm, or 2 mm).

All scans were reconstructed with −50% slice gap (see Methods: MRI Acquisition). Slice-thickness testing (1.0–2.0 mm) occurred mid-study with 13-14 sessions per tested value; most sessions used 2.0 mm (n = 98) or 1.0 mm (n = 56) acquisitions.

#### Diffusion tensor imaging

54 DTI sessions were acquired using a consistent protocol (32 directions, b-values 0 and 800 s/mm², 2 mm isotropic, 60 or 75 slices). Diffusion data are located in session-specific dwi/ subdirectories:

sub-001_ses-YYYYMMDDNN(std)_dwi.nii.gz

Slice count variation (60 vs 75) reflects minor field-of-view adjustments and was not considered significant for protocol categorization. No reversed phase-encoding acquisitions or field maps were obtained; data are therefore provided without susceptibility distortion correction.

### Session-level scan logs

Each session directory contains a *_scans.tsv file listing all acquisitions within that session with their filenames and volume counts. Variable definitions are provided in accompanying JSON sidecars (e.g., sub-001_ses-2024112001_scans.json).

### Source data

**Raw behavioral logs** (sourcedata/sub-001_messages_raw.tsv): Complete unprocessed export from the private Telegram chat used for daily logging throughout the study. This file contains all sleep, caffeine, alcohol, exercise, and general comment entries with original timestamps and shorthand notation codes. Variable definitions and shorthand conventions are documented in the accompanying JSON sidecar. These raw logs are not subject to BIDS validation but are provided for full transparency and reproducibility.

**Pre-session assessments** (sourcedata/assessments/sub-001_pre_session_PVT_PANAS.tsv): Complete trial-level data from 146 pre-scanning assessment sessions, including raw Psychomotor Vigilance Task (PVT) stimulus timestamps and reaction times, and item-level Positive and Negative Affect Schedule (PANAS) responses in mixed-format JSON logs. This enables validation of browser timing characteristics and alternative scoring approaches beyond the summary metrics provided in sessions.tsv.

### Derivatives

**Quality control metrics** (derivatives/mriqc/): Complete MRIQC v25.1.0 outputs for all functional, structural, and diffusion acquisitions, including per-run JSON files with comprehensive image quality metrics (framewise displacement, DVARS, temporal SNR, tissue contrast measures, artifact detection statistics). The directory structure mirrors the main dataset organization. MRIQC dataset provenance is documented in derivatives/mriqc/dataset_description.json.

**Medication data** (derivatives/medication/):

- sub-001_venlafaxine_doses.tsv: Complete chronological record of 286 dose events spanning the entire study period, including timestamps (ISO 8601 format), dosage amounts, administration method (full capsule, capsule half, or bead counting), raw dose calculations where applicable, and source provenance (hand-logged or synthetically generated for pre-taper baseline). Complete variable definitions are provided in the accompanying JSON sidecar.
- Session-level pharmacological regressors (vfx_recent_dose_mg, vfx_21day_avg_mg, vfx_acute_withdrawal, vfx_withdrawal_burden) are integrated into sub-001_sessions.tsv with derivation methods documented in the JSON sidecar and reproducible code provided in code/calculate_vfx_regressors.py.
  **Lifestyle and behavioral data** (derivatives/):
- sleep/sub-001_sleep_events.tsv: Sleep onset and offset times derived from Telegram logs, with 86% coverage (277 of 321 nights). Missing nights are marked as such; synthetic events for modeling purposes are flagged in the source field.
- alcohol/sub-001_alcohol_events.tsv: Alcohol consumption events with timestamps and quantities (0.5 L beer-equivalents, 5% ABV).
- caffeine/sub-001_coffee_events.tsv: Coffee intake events (timestamps only; approximately equivalent caffeine content per cup).
- exercise/sub-001_exercise_sessions.tsv: Logged intentional workout sessions with concatenated free-text exercise notes using compact shorthand codes (e.g., 15sq = 15 squats, 10 = 10 push-ups), explicitly defined in the accompanying JSON sidecar.
- steps/sub-001_steps_events.tsv: Apple Health export - derived step records from iPhone accelerometry.

All derivative files include comprehensive JSON sidecars with variable definitions, units, derivation methods, and references where applicable.

### Data completeness and coverage

#### Standard protocol coverage

128 sessions over 233 days (February 1 – September 22, 2025) with paired 10-minute eyes-open and 14-minute eyes-closed acquisitions, totaling 51.6 hours of standardized resting-state data.

#### Physiological monitoring

Available for 170 functional runs across 89 sessions including 73 standard protocol sessions (covering 57% of standard protocol runs, 37% overall).

#### Pre-session assessments

PVT completed for 146 sessions (87% of standard protocol, 60% overall); PANAS completed for 131 sessions (80% of standard protocol, 54% overall). Summary metrics are provided in sessions.tsv with complete trial/item-level data preserved in sourcedata.

#### Vigilance state documentation

Stanford Sleepiness Scale ratings available for 384 of 458 functional runs (84% coverage), enabling state-dependent analysis of motion and connectivity patterns across the alertness spectrum.

#### Self-administered scanning

189 of 243 total sessions (78%) were conducted using self-administered protocols without operator presence, including 125 of 128 standard protocol sessions (98%).

### File formats and standards

All neuroimaging data are provided in compressed Neuroimaging Informatics Technology Initiative (NIfTI) format (.nii.gz) with BIDS-compliant JSON sidecars containing acquisition parameters and metadata. Physiological data are compressed tab-separated values (.tsv.gz) with JSON sidecars. All tabular metadata, behavioral logs, and derivative files use uncompressed tab-separated values (.tsv) with accompanying JSON sidecars following BIDS conventions where applicable. The dataset has been validated against the BIDS Validator (version 2.1.1) with no errors.

Complete processing code for deriving all pharmacological, behavioral, and physiological regressors is provided in the code/ directory and released under CC0 license (see Code Availability).

## Technical Validation

### Functional MRI quality assessment

**Fig. 2:**
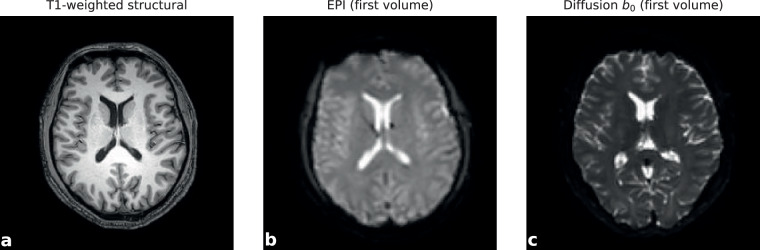
Representative multimodal image quality from a single session. (**a**) T1-weighted structural (1 mm slice thickness, −50% gap reconstruction), (**b**) functional EPI (3 mm isotropic, first volume of eyes-open acquisition), and (**c**) diffusion b₀ image (2 mm isotropic, first volume). All images displayed in neurological orientation (subject’s right on image right) after reorientation to RAS convention. Differences in slice angulation reflect native acquisition geometry; no reslicing was applied. Images illustrate typical quality achieved through self-administered scanning on a clinical 3 T system with standard sequences, without susceptibility distortion correction or spatial smoothing.

Functional image quality was assessed for all 458 runs (230 sessions; 85 hours total) using MRIQC v25.1.0. No susceptibility distortion correction data were acquired. Representative structural, functional, and diffusion images from a single session are shown in Fig. 2, illustrating typical image quality achieved through the self-administered scanning protocol.

### Head motion and vigilance-state dependence

Framewise displacement (FD) distributions indicated consistently low motion across the longitudinal series. Overall, 58 hours of data satisfied a strict threshold of mean FD < 0.2 mm, and 75.4 hours met mean FD < 0.3 mm. When categorized by vigilance state (documented for 89.8% of standard protocol runs, 64% overall), motion varied predictably with arousal level:

- Eyes-open/awake (34.1 h): mean FD = 0.13 mm
- Eyes-closed/awake (10.0 h): mean FD = 0.18 mm
- Partial sleep (7.3 h): mean FD = 0.20 mm
- Frank sleep (17.8 h): mean FD = 0.29 mm

This stratification provides both a high-quality awake subset for conventional connectivity analyses and naturalistic data suitable for studying sleep-related dynamics. Within the standardized protocol (51.6 hours, 128 sessions over 7.5 months), motion quality remained stable throughout the collection period, demonstrating that consistent self-scanning positioning can be maintained over extended timescales.

### Between-session geometric consistency

Positioning reproducibility was quantified by estimating rigid-body realignment parameters between the first volume of each 3iso run and a reference template. Of the 243 total scanning sessions, 189 (78%) were conducted using self-administered protocols without operator presence, including 125 of 128 standard protocol sessions (98%).

During early protocol development and the first half of the study (through June 2025), substantial between-session variability was observed, with translation SD reaching 13.87 mm (z-axis) and rotation SD up to 6.43° (pitch). At least partially, this high variability was attributable to ongoing optimization of scan geometry parameters rather than true head positioning errors - for instance, the elevated pitch SD is inconsistent with plausible positioning mistakes.

Following adoption of a standardized laser-crosshair-to-eyes positioning technique (beginning June 10, 2025) combined with fixed scan geometry parameters, between-session positioning variability decreased substantially:

- Translation SD: x = 1.59 mm, y = 2.59 mm, z = 2.14 mm
- Rotation SD: pitch = 1.11°, roll = 0.62°, yaw = 0.69°

These metrics represent true head positioning variance across sessions under the standardized protocol and demonstrate that sub-3mm, sub-1.5° reproducibility is achievable through self-administered scanning without operator assistance when appropriate anatomical landmarks (laser crosshair aligned to eyes) are consistently employed.

### Temporal signal-to-noise ratio

Run-level temporal SNR (tSNR), calculated as the median voxelwise mean-to-SD ratio from MRIQC outputs, averaged 36 ± 8 (median = 37; range = 12–54), consistent with expectations for single-shot EPI at 3 T with 3 mm isotropic voxels and SENSE = 3 acceleration.

### Comparison of self-administered and operator-assisted acquisitions

All comparisons between self-administered and operator-assisted acquisitions were restricted to 3 mm isotropic EPI runs in order to avoid confounding effects of spatial resolution on framewise displacement (FD) and temporal SNR (tSNR). Of these runs, 361 were self-administered and 54 were operator-assisted.

In an unadjusted comparison, self-administered runs showed higher FD than operator-assisted runs (mean ± SD: 0.187 mm vs 0.137 mm; Welch’s p = 1.5 × 10^−9^; Cohen’s d = 0.72). However, this reflected strong correlations between scanning mode and behavioral state: self-administered sessions were more frequently conducted during periods of reduced alertness. In a regression model controlling for sleep state (categorical Yes/No/A little) and eyes-open vs eyes-closed condition, the self-administration term was not significant (β = 0.019 mm, p = 0.40), whereas sleep (sleep = Yes: β = 0.092 mm, p = 1.53 × 10^−11^) and eyes-open condition (β = −0.041 mm, p = 8.17 × 10^−5^) were the dominant predictors of motion.

A similar pattern was observed for temporal SNR. Unadjusted mean tSNR was slightly lower in self-administered runs (36.6 vs. 38.8; Welch’s p = 0.086, Cohen’s d = −0.27), indicating a weak directional difference. However, this difference was largely accounted for by framewise displacement and behavioral state: in a regression model including FD, sleep, and eyes-open vs. eyes-closed condition, the self-administration term was not significant (β = −0.46, p = 0.85), whereas FD remained a significant predictor (β = −15.5, p = 0.014).

When run duration was included in the model, longer acquisitions were associated with lower tSNR (β = −0.033 per volume, p = 2.51 × 10^−6^), consistent with slow signal drift. Framewise displacement remained a significant predictor (β = −15.2, p = 0.013), whereas self-administration had no detectable effect (p = 0.97).

### Structural MRI quality assessment

T1-weighted image quality was evaluated across 195 scans using MRIQC v25.1.0. Summary metrics indicated consistent image quality with low noise and stable tissue contrast: contrast-to-noise ratio (CNR) = 2.20 ± 0.11 and total SNR = 7.48 ± 0.62. Quality remained stable throughout the 11-month acquisition period.

All T1-weighted scans were reconstructed with vendor-specific −50% slice gap, which interpolates intermediate slices at half-step positions (e.g., 1 mm slices spaced 0.5 mm apart). Approximately half the through-plane resolution is therefore interpolated rather than independently acquired. This reconstruction approach has been used successfully in neuroimaging research for decades and is suitable for standard structural analyses. Users performing voxel-wise analyses may optionally downsample to physically independent spacing before processing (see code/preprocessing/fix_philips_slice_interpolation.py, noting that no thorough testing of this procedure has been done).

### Slice thickness optimization

Slice thickness testing was performed in a controlled manner during the study: 13-14 sessions per tested value (1.25, 1.50, 1.75, 2.00 mm), while the majority used consistent settings (56 sessions at 1.0 mm, 98 sessions at 2.0 mm). Early sessions employed 2 mm slices reconstructed at 1 mm spacing, producing apparently near-isotropic volumes that retained through-plane blurring from interpolation. Subsequent acquisition of true 1 mm slices - approximately doubling slice count and scan time - yielded visibly sharper cortical boundaries and systematically improved metrics: CNR = 2.27–2.32, SNR = 7.6–8.3. This 1 mm protocol was adopted as the final standard for near-isotropic resolution suitable for morphometric and registration analyses.

### Diffusion MRI quality assessment

Diffusion-weighted imaging quality was evaluated across all 54 sessions using MRIQC v25.1.0. All acquisitions employed consistent parameters: single-shot spin-echo EPI, 32 directions, b-values of 0 and 800 s/mm², 2 mm isotropic resolution, SENSE = 2.0.

Signal-to-noise ratios remained consistent across the 11-month period (median SNR = 7.7, IQR = 1.7), and noise estimates showed tight distributions without evidence of systematic drift. The median neighboring-direction correlation (NDC = 0.59, IQR = 0.17) was somewhat lower than typically observed in full-brain multi-shell acquisitions, but this was primarily attributable to partial brain coverage rather than motion or noise. In later sessions using extended 75-slice coverage (September 2025 onward), the median NDC increased to 0.74, comparable to modern single-shell benchmarks. Because NDC is computed over whole-brain masks, incomplete coverage systematically lowers spatial correlations across diffusion volumes. Visual inspection confirmed stable signal and artifact levels consistent with expectations for single-shell clinical DTI.

No field maps or reversed phase-encoding acquisitions were obtained; diffusion data are provided without susceptibility distortion correction.

### Scanner stability and longitudinal quality trends

No dedicated phantom acquisitions were performed during the study period, limiting the ability to distinguish hardware-related drift from changes in participant positioning, protocol refinement, or physiological factors. Functional tSNR showed a modest positive trend over time (≈ + 0.9 units/month in eyes-open runs), while anatomical quality metrics exhibited thickness-dependent temporal patterns. These trends may reflect a combination of scanner-related variability, improvements in self-positioning technique, and vigilance-related effects.

Run-level analyses indicate that tSNR variation is primarily associated with framewise displacement and run duration, both of which covary with vigilance state during long acquisitions. After accounting for these factors, scanning mode does not independently predict tSNR. These relationships are descriptive and do not imply underlying biological mechanisms. Because no phantom data were acquired, residual contributions from scanner drift or slow hardware changes cannot be excluded.

Observed CNR and tSNR values fall within ranges commonly reported for clinical 3 T systems, although hardware-related contributions to longitudinal variability cannot be quantitatively bounded in the absence of phantom data. Session-level quality metrics (tSNR, FD, CNR) are provided in derivatives/mriqc/ to enable sensitivity analyses and quality-based data subsetting.

### Temporal co-occurrence of study factors

Quantitative inspection confirmed that several study variables evolved nearly monotonically with time. Across n = 243 sessions, venlafaxine dose metrics were almost perfectly correlated with study day (Spearman ρ = −0.97 to −0.98, *p* < 10^−100^), reflecting the planned taper. Self-administration experience increased correspondingly (ρ = 1.00, *p* < 10^−200^). Photoperiod (daylight duration) showed a moderate positive correlation with study day (ρ = 0.31, *p* = 1.9 × 10^−8^) and with the withdrawal-burden metric (ρ = −0.65, *p* = 2.6 × 10^−18^), illustrating the seasonal overlap with the taper period. After removing the linear time trend, residual venlafaxine dose and photoperiod were inversely related (*partial* r = −0.43, *p* = 3.2 × 10^−11^), consistent with lower doses coinciding with longer daylight periods.

These findings indicate that pharmacological state, seasonal change, and self-scanning experience are strongly time-locked and cannot be separated analytically within this dataset. The study is therefore best interpreted as a naturalistic longitudinal observation, demonstrating the feasibility of capturing pharmacological and seasonal transitions using self-administered clinical MRI rather than providing controlled pharmacological contrasts.

### Pre-scan behavioral measures

#### Psychomotor vigilance task

PVT performance (n = 146 sessions) indicated consistently alert pre-scan states: median reaction times averaged 299 ± 18 ms (IQR: 287–312 ms), consistent with alert performance on web-based vigilance tasks. The narrow distribution indicates stable attentional capacity across the study, with slower mean compared to hardware-based PVT implementations (~250 ms) attributable to expected browser timing overhead. Lapse counts (RT > 500 ms) were low overall (mean: 0.9 ± 1.1; median: 1; range: 0–5), with 114 of 146 sessions (78%) showing ≤ 1 lapse during the 3-minute task. Only 13 sessions (9%) showed ≥ 3 lapses.

#### PANAS mood assessments

PANAS scores (n = 131 sessions with complete responses) showed stable baseline mood with within-subject variability. Positive affect averaged 28.8 ± 4.5 (IQR: 26–33), while negative affect averaged 16.9 ± 3.5 (IQR: 14–18). These measures are provided as behavioral annotations without causal interpretability within this dataset.

## Usage Notes

### Dataset limitations

#### Single-subject and temporally confounded design

This dataset was acquired from a single participant over an 11-month period during which several key factors evolved nearly monotonically with time, including antidepressant dose, seasonal photoperiod, and self-scanning experience and workflow optimization. These variables are therefore strongly collinear and cannot be analytically disentangled within an N = 1 design. Importantly, the dataset should be interpreted as a methodological and infrastructural resource rather than as evidence for specific neurobiological effects. The longitudinal context represents descriptive co-occurrence rather than a controlled experimental manipulation.

#### Absence of susceptibility distortion correction

No field maps or reversed phase-encoding acquisitions were obtained, functional and diffusion MRI data are therefore provided without susceptibility distortion correction. This limits absolute spatial accuracy and anatomical localization, particularly in ventral frontal and temporal regions. Because acquisition geometry and phase-encoding direction were consistent across standardized sessions, susceptibility distortions are expected to be systematic over time, supporting longitudinal consistency while constraining spatial interpretability.

#### Diffusion MRI protocol constraints

Diffusion data were acquired using a single-shell protocol with 32 directions at b = 800 s/mm². While sufficient for basic tensor-level estimation and longitudinal consistency assessments, these data are not optimized for advanced tractography, multi-compartment modeling, or microstructural inference. Diffusion data should therefore be regarded primarily as a methodological and feasibility resource rather than a comprehensive diffusion imaging dataset.

#### Subjective vigilance and behavioral measures

Vigilance state during scanning was assessed using self-reported Stanford Sleepiness Scale ratings and retrospective estimates of sleep occurrence. These measures provide coarse, subjective approximations of arousal state and may be affected by recall bias. Similarly, pre-scan behavioral assessments (PVT, PANAS) were administered via a web-based interface and reflect relative within-subject variation rather than laboratory-grade psychometric precision. Users are encouraged to supplement or replace these measures with objective proxies derived from physiological recordings or motion characteristics where appropriate.

#### Absence of independent scanner drift calibration

No dedicated phantom acquisitions were performed during the study period. Consequently, residual scanner drift or slow hardware changes cannot be independently separated from physiological, behavioral, or procedural factors. Although observed longitudinal trends in image quality metrics fall within expected ranges for clinical 3 T systems, the absence of phantom data represents a limitation for attributing long-term signal changes exclusively to biological or behavioral sources.

### Getting started with the dataset

For users seeking the core standardized data, sessions marked with the std suffix (n = 128 sessions over 7.5 months) contain paired 240-volume eyes-open and 340-volume eyes-closed runs (acq-3isoEOstd and acq-3isoECstd), totaling 51.6 hours. The dataset-wide summary file derivatives/sub-001_func_runs.tsv includes stdEO and stdEC boolean columns for efficient filtering.

Key metadata locations:

- sub-001/sub-001_sessions.tsv: session-level metadata and covariates (including pharmacological regressors and summary behavioral measures).
- derivatives/sub-001_func_runs.tsv: run-level metadata for all fMRI runs (protocol labels, volume counts, vigilance annotations and selected QC measures).
- derivatives/mriqc/: per-run MRIQC outputs for functional, structural, and diffusion acquisitions.

### Quality control recommendations

Run-level quality metrics from MRIQC are provided in derivatives/mriqc/. Users can apply flexible quality thresholds based on their analysis approach: 58 hours meet strict criteria (mean FD < 0.2 mm), 75.4 hours meet moderate criteria (mean FD < 0.3 mm). Motion varies systematically with vigilance state, enabling both stringent connectivity analyses using high-quality awake data and investigation of state-dependent dynamics across the arousal spectrum.

### Protocol evolution and cross-phase comparisons

The scanning protocol evolved over 11 months: a 3-month development phase optimized self-scanning procedures, followed by 7.5 months of standardized data collection (128 sessions marked with std suffix). T1-weighted slice thickness testing (1.0–2.0 mm) occurred mid-study; most sessions used 2.0 mm (n = 98) or 1.0 mm (n = 56) acquisitions. Users performing cross-phase comparisons should account for these protocol changes using the metadata provided in the BIDS sidecars and session/run summary TSV files.

## Data Availability

All data described in this Data Descriptor are publicly available on OpenNeuro (dataset accession number: ds007328)^7^. The dataset follows the Brain Imaging Data Structure (BIDS) v1.9.0 specification and includes raw MRI data, derivatives, and sourcedata as detailed in the *Data Records* section. All materials are released under the Creative Commons CC0 1.0 Public Domain Dedication, as specified in the dataset metadata, and have been validated with the BIDS Validator (v2.1.1).

## Code availability

Processing scripts for SCANPHYSLOG conversion, PVT/PANAS scoring, and pharmacokinetic modeling are included in the code/ directory and released under CC0. PVT/PANAS testing interface is available at https://eugenpt.github.io/ep_fmri_tests/pvt.html.

## References

[1] Van Essen, D. C. *et al*. The WU-Minn Human Connectome Project: An overview. *Neuroimage* **80**, 62–79, 10.1016/J.NEUROIMAGE.2013.05.041 (2013). 23684880 10.1016/j.neuroimage.2013.05.041 PMC3724347

[2] Miller, K. L. *et al*. Multimodal population brain imaging in the UK Biobank prospective epidemiological study. *Nat. Neurosci*. **19**, 1523–1536, 10.1038/NN.4393;TECHMETA (2016). 10.1038/nn.4393 PMC5086094 27643430

[3] Poldrack, R. A. *et al*. Long-term neural and physiological phenotyping of a single human. *Nat. Commun*. **6**, 1–15, 10.1038/NCOMMS9885;TECHMETA (2015). 10.1038/ncomms9885 PMC4682164 26648521

[4] Choe, A. S. *et al*. Reproducibility and Temporal Structure in Weekly Resting-State fMRI over a Period of 3.5 Years. *PLoS One* **10**, e0140134, 10.1371/JOURNAL.PONE.0140134 (2015). 26517540 10.1371/journal.pone.0140134 PMC4627782

[5] Gulban, O. F. *et al*. poldracklab/pydeface: v2.0.0. 10.5281/ZENODO.3524401.

[6] Watson, D., Clark, L. A. & Tellegen, A. Development and Validation of Brief Measures of Positive and Negative Affect: The PANAS Scales. *J. Pers. Soc. Psychol.* **54**, 1063–1070, 10.1037/0022-3514.54.6.1063 (1988). 3397865 10.1037//0022-3514.54.6.1063

[7] Petrovskiy, E. Dense longitudinal single-subject multimodal MRI dataset acquired via self-administered scanning, *OpenNeuro*, 10.18112/openneuro.ds007328.v1.0.0 (2025). 10.1038/s41597-026-06879-z PMC13035833 41723198

